# TIGIT expression in extrahepatic cholangiocarcinoma and its impact on CD8 + T cell exhaustion: implications for immunotherapy

**DOI:** 10.1038/s41419-025-07388-4

**Published:** 2025-02-12

**Authors:** Tengqian Tang, Wenhao Wang, Lang Gan, Jie Bai, Dehong Tan, Yan Jiang, Ping Zheng, Weijun Zhang, Yu He, Qianfei Zuo, Leida Zhang

**Affiliations:** 1 Department of Hepatobiliary Surgery, First Affiliated Hospital, Army Medical University, Chongqing, 400038 PR China; 2 College of Pharmacy, Henan University, Kaifeng, 475001 PR China; 3 Department of Microbiology and Biochemical Pharmacy, College of Pharmacy, Army Medical University, Chongqing, 400038 PR China

**Keywords:** Cancer metabolism, Cancer prevention

## Abstract

Extrahepatic cholangiocarcinoma (ECCA) is a malignant tumor. The precise role of T-cell immunoreceptor with Ig and ITIM domains (TIGIT), an emerging immunosuppressive receptor, in ECCA, and its impact on CD8+ T cell exhaustion (Tex) remains unclear. We performed single-cell RNA sequencing (scRNA-seq) to characterize tumor-infiltrating lymphocytes (TILs) isolated from ECCA. We found that TIGIT was significantly overexpressed in TOX+CD8 T cells. Tissue microarray and immunohistochemistry staining demonstrated that increased TIGIT expression was associated with poorer patient survival. Flow cytometry analysis revealed that TIGIT+CD8+ T cells exhibited decreased TNF-α, IFN-γ, and TCF-1 expression, accompanied by elevated PD-1 and TIM-3 expression compared to TIGIT−CD8+ T cells. In the patient-derived xenograft (PDX) model, the anti-TIGIT treatment group demonstrated reduced tumor weight, enhanced CD8 frequency, and an increased IFN-γ proportion compared to the PBS treatment group. The TIGIT antibody-treated group exhibited a notably higher fraction of GRZB, and anti-TIGIT treatment led to elevated TCF-1 protein levels and decreased protein levels of TOX1 and NR4A1. Moreover, TIGIT+CD8 T cells from TILs appear to be in a state of exhaustion with low potential killing capacity in ECCA, as shown by scRNA-seq. Taken together, the present study underscores the significant role of TIGIT in ECCA, contributing to T cell exhaustion and a compromised CD8+ T cell immune response. Targeting TIGIT presents a promising therapeutic avenue to enhance the CD8+ T-cell response, thereby potentially improving ECCA therapeutic benefits.

## Introduction

Biliary tract carcinoma (BTC) is a relatively uncommon malignancy, yet its prevalence has been steadily increasing over the past 30 years [1]. BTC encompasses various subtypes, including intrahepatic cholangiocarcinoma (ICC, constituting 10–20% of all cases), extrahepatic cholangiocarcinoma (ECCA) with perihilar (PCC, 50–60%), distal (DCC, 20–30%), and gallbladder carcinoma (GBC, 0.4–3.8%) [2]. ECCA typically lacks distinctive clinical symptoms in its early stages, leading to 70–80% of patients being diagnosed at advanced disease stages, often accompanied by infiltration and metastasis. The concealed location of ECCA lesions poses challenges for surgical resulting in, leading to a low rate of successful radical resection. While the 5-year survival rate after radical resection is ~16.5%, it drops to <10% for unresectable cases, indicating a dire prognosis [3]. Consequently, the identification and validation of cancer drivers that can serve as potential therapeutic targets become imperative.

Immunotherapy has emerged as a promising avenue for treating cancer, harnessing immune system to combat malignant cells. As new findings emerge, Tex poses a hurdle in to effective immunotherapy, necessitating strategies to reverse Tex and restore T cell functionality [4]. As is well known, TOX is one of the most important transcription factors in exhaustion T cell, while TCF-1 has surfaced as a crucial transcription factor associated with positive outcomes in melanoma patients undergoing checkpoint blockade therapy [5]. This underscores the significance of TCF-1 in maintaining CD8 T cell activity in the presence of persistent viral or tumor antigens. Immune checkpoint blockade, such as targeting PD-1, Lag-3, Tim-3, has demonstrated potential in reinvigorating tumor-infiltrating cell function and enhancing immunotherapy results [4]. TIGIT, an immune checkpoint protein expressed on T cells and NK cells [6], exerts immunosuppressive effects by engaging with CD155 on target or antigen-presenting cell [6]. Moreover, TIGIT expression has been associated with tumor growth in diverse cancers [7], including chronic lymphocytic leukemia and acute myeloid leukemia (AML) [8]. To our knowledge, no TIGIT has been targeted in ECC therapy to date. Hence, targeting TIGIT presents a new approach in to ECCA treatment.

This study aimed to collect 302 human ECCA tissue samples, which is one of the largest such collections in the world, in order to investigate the expression of TIGIT and its impact on CD8+ Tex in patients with ECCA. Additionally, the potential efficacy of a TIGIT antibody as an immunotherapeutic agent in ECCA will be evaluated. The outcomes of this research hold the potential to pave the way for innovative immunotherapy strategies, providing renewed optimism for enhancing therapeutic effectiveness and survival rates in ECCA patients.

## Materials and methods

### Human tissue specimens

Human extrahepatic cholangiocarcinoma (ECCA) samples were obtained from 302 patients treated at the Department of Hepatobiliary Surgery, First Affiliated Hospital of Army Medical University (Chongqing, China) between 2010 and 2021. Informed consent was obtained from all participants, and the study was approved by the regional ethics committee and institutional review board.

### Tissue microarrays

Tissue microarrays (TMAs) were constructed by Wuhan Servicebio Biotechnology Co. Immunohistochemical (IHC) analysis was performed on sections from 89 ECCA samples for TIGIT protein expression. Slides were dewaxed, rehydrated, treated with H_2_O_2_ to block endogenous peroxidase activity, and underwent antigen retrieval in EDTA buffer. Sections were incubated with primary anti-TIGIT antibodies (1:300) overnight at 4 °C, followed by incubation with a secondary antibody, staining with hematoxylin, and mounting for analysis.

### Immunohistochemistry

Formalin-fixed, paraffin-embedded human ECCA tissues were processed for IHC. Endogenous peroxidase was blocked, and sections were incubated with goat serum to prevent non-specific binding. Primary antibodies against CD3 and TIGIT were applied overnight at 4 °C. The next day, slides were treated with species-specific HRP-conjugated secondary antibodies, followed by DAB staining.

### Immunofluorescence

Tissue sections were fixed, permeabilized, and subjected to antigen retrieval. After blocking with bovine serum albumin (BSA), tissues were incubated overnight with anti-CD8 and anti-TIGIT antibodies. After washing, fluorescent secondary antibodies were applied, and nuclei were stained with DAPI. Samples were mounted for fluorescent imaging.

### Flow cytometry

Human and mouse ECCA tissues were dissociated into single-cell suspensions using collagenase. Cells were labeled with antibodies targeting CD8, PD-1, IFN-α, TNF-γ, granzyme B (GRZB), and TCF-1 (Biolegend). Flow cytometry analysis was performed to evaluate immune markers.

### Survival analysis

Survival analysis was conducted on 289 ECCA patient samples (after excluding 13 cases lost to follow-up) using Kaplan–Meier curves and the log-rank test. Cox proportional hazards models were employed to assess the significance of TIGIT expression levels (low vs. high).

### Patient-derived xenograft (PDX) model

PDX models were established using tumor tissues from ECCA patients, which were implanted subcutaneously into SCID mice. Tumor growth was monitored biweekly, and treatment groups received either PBS or anti-TIGIT therapy. Tumor volume was calculated, and animals were euthanized when tumor burden or body weight loss exceeded predefined limits. Six mice in each group were randomly selected for follow-up flow cytometry analysis. Blinding was done. Ethical approval for all animal experiments was obtained from the Animal Ethical and Experimental Committee of the Army Medical University.

### Western blotting

Mouse tissue samples were lysed, and protein concentrations were measured using the BCA assay. SDS–PAGE was followed by membrane transfer and blocking. Membranes were incubated with primary antibodies (TCF-1, TOX1, NR4A1, PTPN2, GAPDH) and scanned using the Odyssey imager.

### Single-cell RNA sequencing

Tumor-infiltrating lymphocytes (TILs) were isolated from ECCA tissues following digestion with a Tumor Dissociation Kit. Single-cell RNA libraries were prepared using the 10x Genomics platform and sequenced on an Illumina HiSeq X Ten with 150-bp paired-end reads. The sequences data was added in supplementary information.

### Statistical analyses

Statistical analyses were performed using GraphPad Prism 9.0. Group comparisons were conducted via analysis of variance (ANOVA), and survival curves were assessed using Kaplan–Meier analysis with log-rank tests. A *p*-value < 0.05 was considered statistically significant.

## Result

### Clinicopathological profiles of ECCA patients

A total of 302 ECCA patients were included in this study, with 116 (38.5%) being male and 186 (61.5%) being female, and a median age of 61 years. Among these patients, 252 (83.4%) were diagnosed with perihepatic cholangiocarcinoma, and 50 (16.6%) with distant cholangiocarcinoma. Surgical interventions were performed on 280 patients (92.8%), including 141 (46.8%) who underwent left hepatectomy, 60 (19.9%) who underwent right hepatectomy, 30 (9.8%) who underwent mesohepatectomy, 46 (15.2%) who underwent pancreaticoduodenectomy, 3 (7.2%) who underwent hepatopancreoduodenectomy, and 22 (7.2%) who underwent palliative surgery. Regarding tumor differentiation, 40 (13.3%) patients had highly differentiated tumors, 189 (62.6%) had moderately differentiated tumors, and 73 (24.1%) had poorly differentiated tumors.

Among patients with perihepatic cholangiocarcinoma, the Bismuth-Corlette classification revealed no type I cases, 34 (13.6%) type II cases, 59 (23.6%) type IIIa cases, 85 (33.6%) type IIIb cases, and 74 (29.2%) type IV cases. Median bilirubin level was 137.1 μmol/L, median albumin level was 34.9 g/L, and median CA19-9 level was 102.6 ku/L. Tube drainage was absent in 142 patients (47.1%), while 95 (31.3%) underwent percutaneous transhepatic cholangiography (PTC), and 52 (17.2%) underwent endoscopic retrograde cholangiography (ERC). Both PTC and ERC were performed in 13 patients (4.4%). The disease-free survival time for patients with perihepatic cholangiocarcinoma was 12 months, while the overall survival time was 20 months. A summary of clinicopathological profiles is provided in Table 1.

**Table 1:** Patient clinicopathologic profiles.

Variable	Cholangiocarcinoma patients (*n* = 302)
Age, md years (range)	61 (30–84)
*Sex*, *n* *(%)*	
Female	116 (38.5%)
Male	186 (61.5%)
Radical operation, *n* (%)	280 (92.8%)
Left hepatectomy	141 (46.8%)
Right hepatectomy	60 (19.9%)
Mesohepatectomy	30 (9.8%)
Pancreaticoduodenectomy	46 (15.2%)
Hepatopancreoduodenectomy	3 (1.1%)
Palliative operation, *n* (%)	22 (7.2%)
*Diagnosis*, *n* *(%)*	
Perihilar cholangiocarcinoma	252 (83.4%)
Distal cholangiocarcinoma	50 (16.6%)
*Tumor grade*, *n* *(%)*	
Well differentiated	40 (13.3%)
Moderately differentiated	189 (62.6%)
Poorly differentiated	73 (24.1%)
*Perihilar cholangiocarcinoma* (*n* = 252), *Bismuth-Corlette classification*, *n* (%)	
I	0
II	34 (13.6%)
IIIa	59 (23.6%)
IIIb	85 (33.6%)
IV	74 (29.2%)
Bilirubin μmol/L, md (range)	137.1 (9.1–562.7)
Albumin g/L, md (range)	34.9 (25.1–51.4)
CA19-9 Ku/L, md (range)	102.6 (1.5–800)
*Biliary drainage*, *n* *(%)*	
No drainage	142 (47.1%)
Percutaneous transhepatic cholangiography (PTC)	95 (31.3%)
Endoscopic retrograde cholangiography (ERC)	52 (17.2%)
PTC and ERC	13 (4.4%)
Perihilar cholangiocarcinoma Disease-free survival, md months (range)	12 (0–74)

*CA19-9* carbohydrate antigen 19-9.

### High-resolution immune landscape of ECCA by single-cell RNA sequencing

**Fig. 1:**
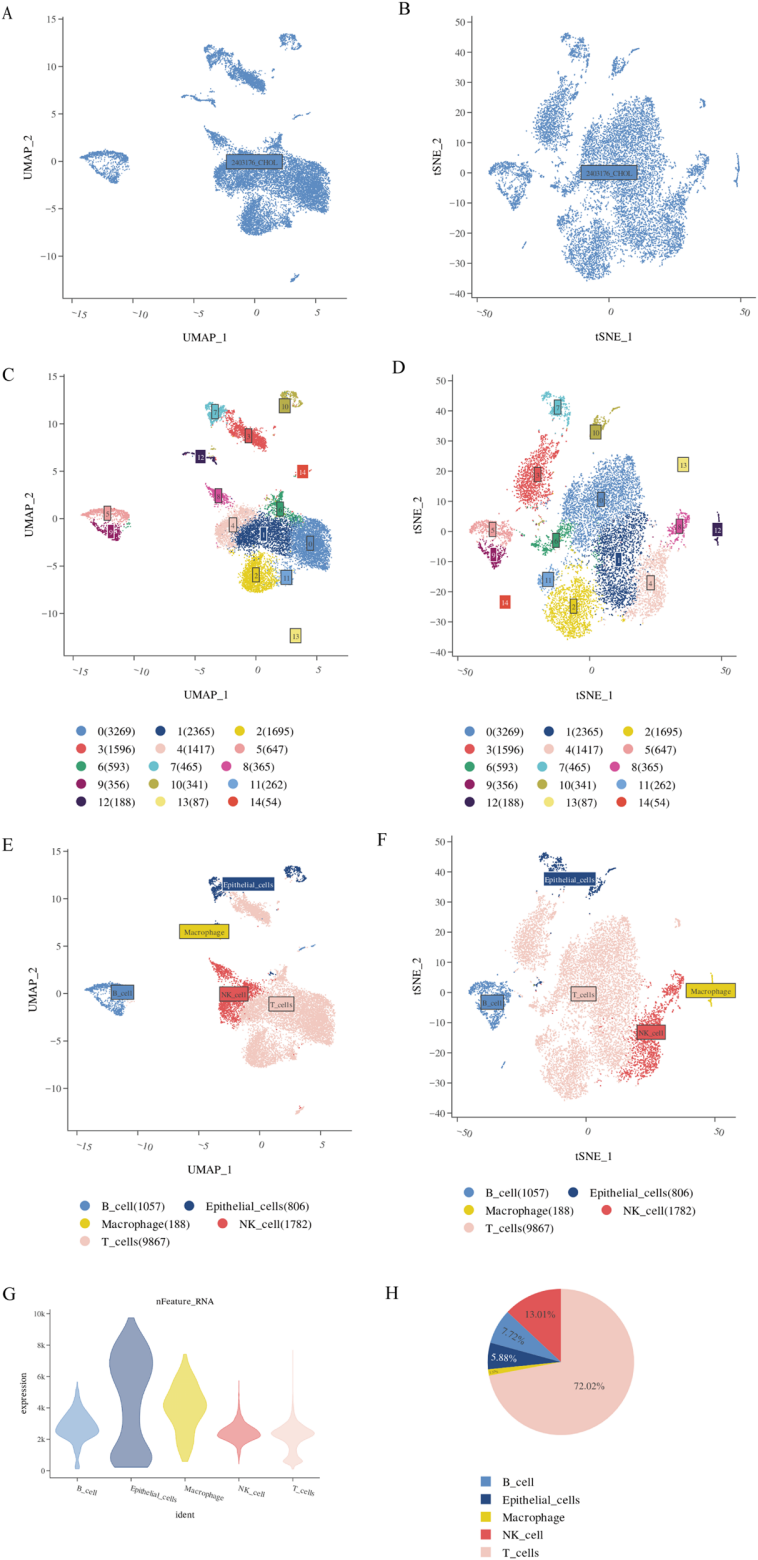
Basic information on the data and the integration of TIL cells. **A** Uniform manifold approximation and projection (UMAP) visualizations of TIL cells. **B** TSNE visualizations of TIL cells. **C** The distribution of each cell population in the UMAP map obtained by corresponding set parameters. **D** The distribution of each cell population in the TSNE map obtained by corresponding set parameters. **E** After dimensionality reduction, annotate UMAP results (resolution.0.5_d30). **F** After dimensionality reduction, annotate TSNE results (resolution.0.5_d30). **G** The total amount of gene expression in a single cell by nFeature_RNA. **H** Pie chart of annotated cell types in all cells.

To characterize the immune cells in ECCA we applied scRNA-seq methods to study tumor-infiltrating lymphocytes (TIL) cells isolated from tumors. After initialization, a uniform threshold was set for filtering, removing low-quality cells, and reserving 13,000 qualified cells for subsequent analysis. The samples with a filtered cell number of final Num were integrated and were gathered together to ensure that different samples with the same cell type were in similar positions in the dimensionality reduction map (Fig. 1A, B). Cell annotation is an important part of single-cell analysis, and subsequent analysis is carried out on the basis of annotated cell types. Manually check and annotate marker genes corresponding to cell types in published articles using singleR’s built-in reference dataset. The annotation results are shown in Fig. 1C, D. For further dimension reduction annotation, the main cell subsets in TIL cells include T cells, NK cells, B cells and macrophages (Fig. 1E, F). Interestingly, the total of T cells was 9867, accounting for 72.02% (Fig. 1E–H). These results suggest that T cells are the most abundant cell subpopulation in TILs and may play an important role in the tumor environment of ECCA.

### The immune checkpoint molecule TIGIT was significantly overexpressed in the TOX+CD8 T cells

**Fig. 2:**
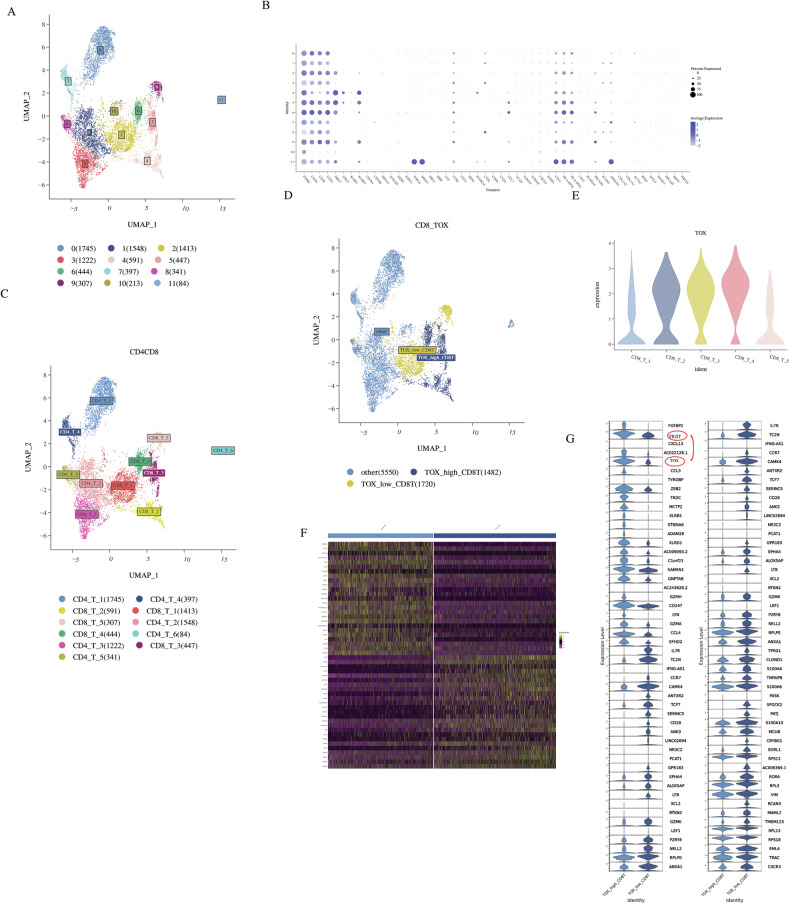
Basic information of the data and the integration of TOX+CD8 T and TOX−CD8 T cells. **A** Uniform manifold approximation and projection (UMAP) visualizations of T cells. **B** Large group marker analysis. (The size of the dot represents the proportion of cells expressing the gene in the group, and the color depth represents the average expression of the gene in the group.) **C** UMAP visualizations of CD4+T and CD8+T cells. **D** UMAP visualizations of TOX+CD8 T and TOX−CD8+T cells. **E** The expression of TOX in different CD8+T cell subsets. **F** Heat maps show the top50 differential genes between TOX+CD8 T and TOX−CD8+T cells. **G** The violin diagram shows the top50 differential genes between TOX+CD8 T and TOX−CD8+T cells.

To further clarify the function of T cell subsets, we annotated the subsets according to T cell-related makers (PTPRC, CD3D, CD3E, CD3G, NKG7, eg) (Fig. 2A, B). Further clustering CD4 cells and CD8T cells, CD4 T cells can be subdivided into six clusters, CD8T cells can be subdivided into five clusters (Fig. 2C, D), and the expression of TOX mainly clustered in CD8 clusters 1–5 (Fig. 2E). As well known, TOX is one of the most important transcription factors in the exhaustion T cell. Then, Next, we analyzed differential gene expression in the transcriptomic profiles of TOX+CD8 and TOX−CD8 subgroups. The results showed that ZEB2(CD247, SAMSN1) was highly expressed in TOX+CD8 subgroups. Moreover, we found novel immune checkpoint molecule TIGIT was significantly overexpressed in the TOX+CD8 T cells (Fig. 2F, G). These results suggest that the high expression of TIGIT in ECCA may be involved in T cell exhaustion.

### Association of high TIGIT expression with reduced survival in ECCA Patients

**Fig. 3:**
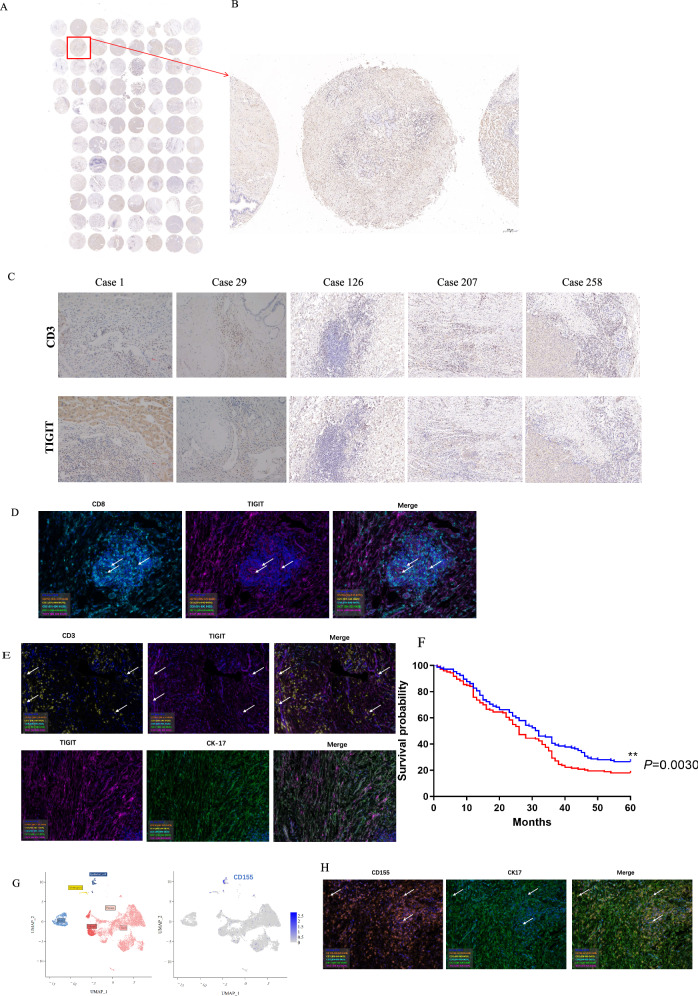
Association of high TIGIT expression with reduced survival in ECCA patients. **A** Immunohistochemistry staining in tissue chip of TIGIT protein expression in extrahepatic cholangiocarcinoma. *n* = 89. **B** Immunohistochemical detection of TIGIT in paraffin section. **C** TIGIT and CD3 were expressed in extrahepatic cholangiocarcinoma by Immunohistochemistry staining. **D** Immunofluorescence detection of TIGIT and CD8 expression in T cell surface. **E** double immunostaining of TIGIT with CD3 and CK-17. **F** TIGIT in ECC survival analysis. *n* = 289, **P = 0.003. **G** Cell types expressing CD155 by RNA sequencing. **H** CD155 staining with CK-17 via double immunofluorescence in human ECCA tissues.

An analysis of tissue microarrays involving randomly selected samples from 89 ECC patients demonstrated positive TIGIT expression in these specimens (Fig. 3A, B). The immunohistochemistry staining of CD3 and TIGIT for tissue specimens of five ECC patients (cases 1, 29, 126, 207, and 258) are depicted (Fig. 3C). Double immunostaining of TIGIT with CD8, CD3 revealed co-localization of TIGIT and CD8 on the cell surface (Fig. 3D, E).

Survival analysis outcomes indicated that ECCA patients with low TIGIT expression exhibited a notably higher survival rate (*P* < 0.05 for TIGIT low vs. TIGIT high; Fig. 3F). Specifically, the median survival period for ECCA patients with low TIGIT expression was 32 months, while it stood at 26 months for ECCA patients with high TIGIT expression. These findings suggest a potential regulatory role of TIGIT in the immune response of ECCA patients, with elevated TIGIT expression potentially linked to unfavorable prognosis within this patient cohort.

For TIGIT exerts immunosuppressive effects by engaging with CD155. We analyzed the cell types expressing CD155 by RNA sequencing and stained CD155 (Fig. 3G) with the specific cell marker (CK-17) via double immunofluorescence in human ECCA tissues (Fig. 3H). The results showed that CD155 was mainly expressed in epithelial cells.

### TIGIT as a potential surface marker for T cell exhaustion in ECCA

**Fig. 4:**
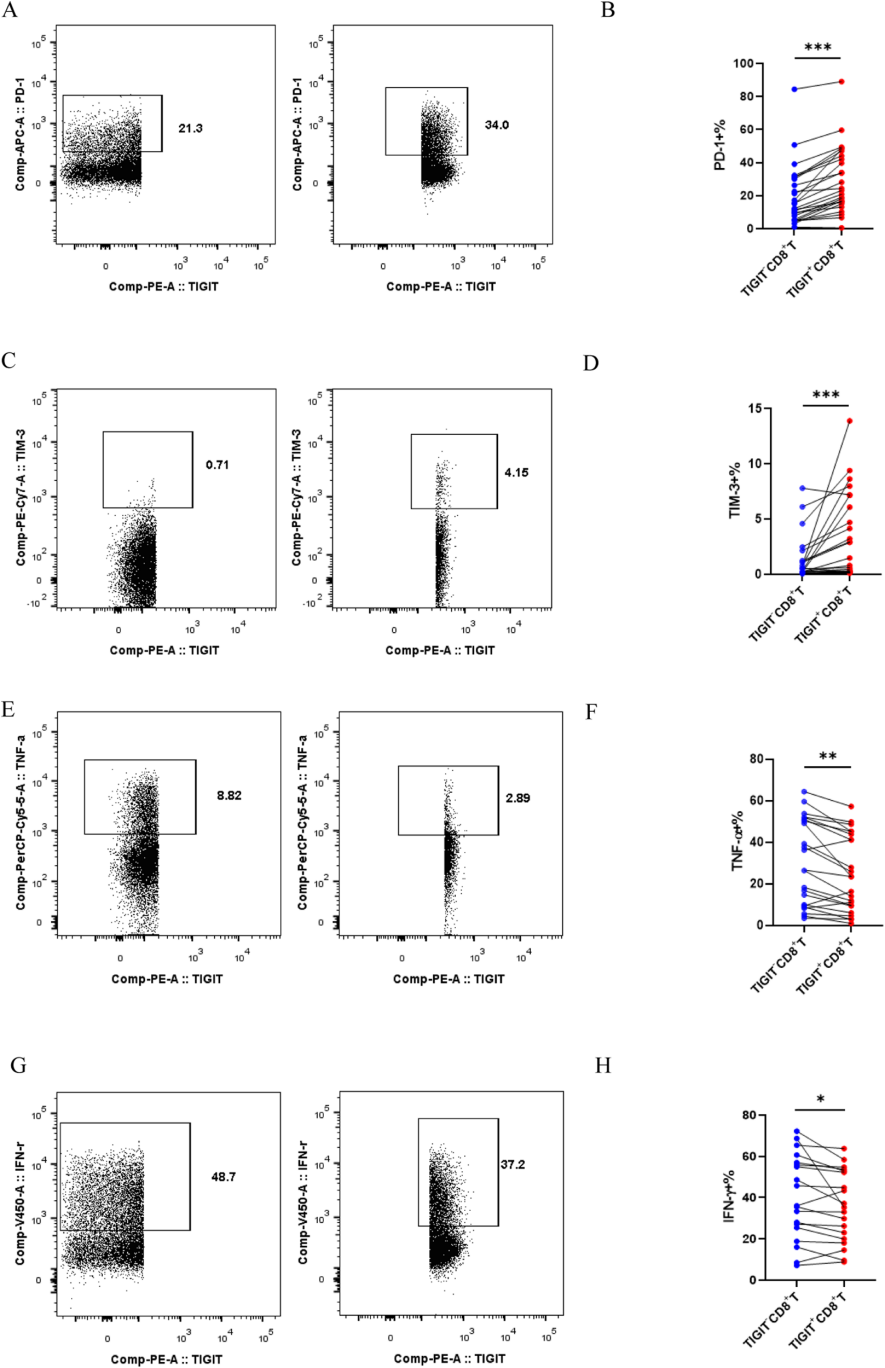
TIGIT as a potential surface marker for T cell exhaustion in ECCA. **A** and **B** Flow cytometric analysis of immunological checkpoint PD-1 expression in TIGIF-CD8+ and TIGIF+CD8+T cell subsets. **C** and **D** Flow cytometric analysis of immunological checkpoint TIM-3 expression in TIGIF−CD8+ and TIGIF+CD8+T cell subsets. **E** and **F** Flow cytometric analysis of the cytokine IFN-α expression in TIGIF-CD8+ and TIGIF+CD8+T cell subsets. **G** and **H** Flow cytometric analysis of the cytokine TNF-γ expression in TIGIF−CD8+ and TIGIF+CD8+T cell subsets. TIGIF-CD8+ and TIGIF+CD8+T cell subset levels were compared by paired Student’s *t*-test, *n* = 26, *P < 0.05, **P < 0.01, *P* < 0.001***.

The utilization of flow cytometry to analyze T cells in ECC patients revealed noteworthy findings. TIGIT-positive (TIGIT+) CD8 T cells exhibited notably higher expression levels of programmed death-1 (PD-1) and T cell immunoglobulin domain and mucin domain-3 (TIM-3) in comparison to TIGIT-negative (TIGIT-) CD8 T cells (*P* < 0.01 for both comparisons; Fig. 4A–D). Furthermore, TIGIT+CD8 T cells demonstrated decreased levels of tumor necrosis factor (TNF-α) and interferon-gamma (IFN-γ) compared to TIGIT−CD8 T cells (*P* < 0.01 for both comparisons; Fig. 4E–H). These observations suggest that TIGIT may collaborate with other immune checkpoint molecules in inducing CD8 T cell exhaustion, potentially contributing to immune evasion mechanisms within the immune microenvironment of ECCA.

### Reduced expression of TCF-1 in TIGIT+CD8+ T cells from ECCA patients, indicative of Tex

**Fig. 5:**
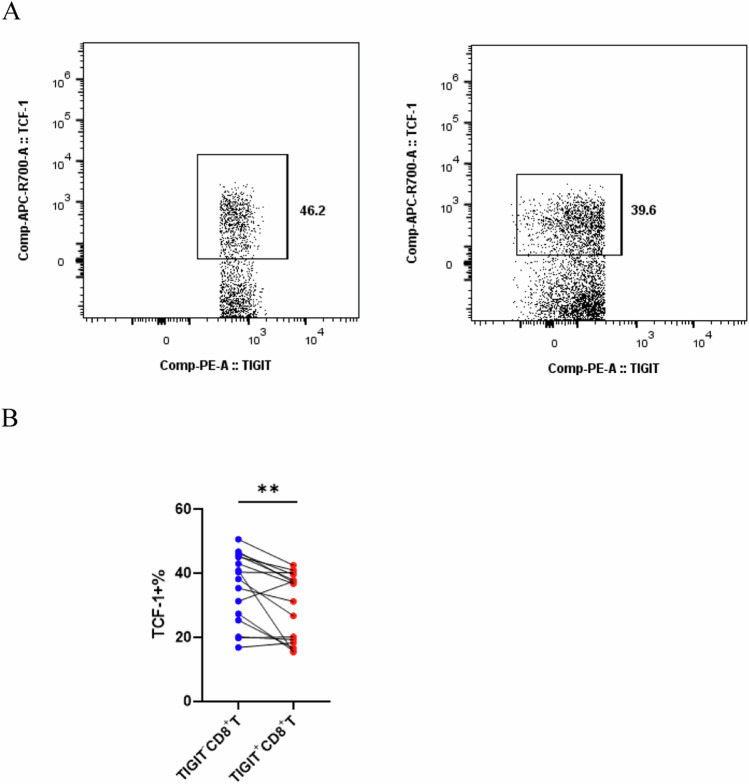
Reduced expression of TCF-1 in TIGIT+CD8+ T cells from ECC patients. **A** and **B** Flow cytometric analysis the expression of the transcription factor TCF-1 in TIGIF-CD8+ and TIGIF+CD8+ T cells. Flowcytometry analysis for TCF-1 transcription factor expression on CD8+ T-cells. TIGIF−CD8+ and TIGIF+CD8+ specific T cell subset levels were compared by paired Student’s *t*-test, **P < 0.01.

To enhance our comprehension of the effect of TIGIT on Tex in ECCA, we investigated the TCF-1 transcription factor. The result showed a reduced proportion of TCF-1 + T cells in the TIGIT+CD8+ T cells compared to the TIGIT−CD8+ T cells (Fig. 5A, B, *p* < 0.05). There are findings, that a negative correlation between TIGIT expression and TCF-1 expression was observed, indicative of Tex.

### Immunotherapeutic effects of TIGIT antibody in the PDX model

**Fig. 6:**
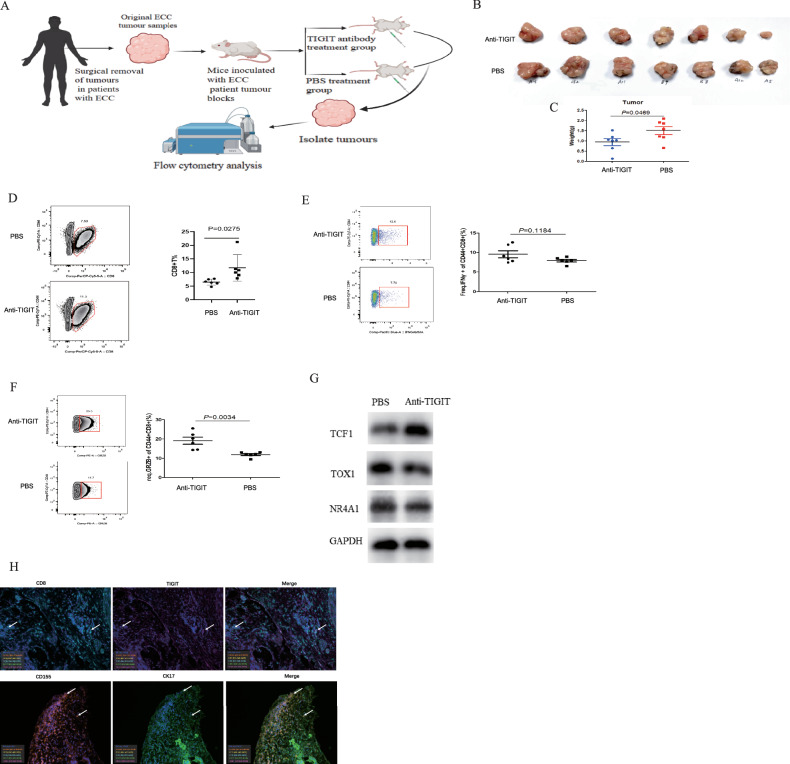
Immunotherapeutic effects of TIGIT antibody in the PDX model. **A** Creating a flowchart of a patient-derived xenograft (PDX) model using the BioRender tool. **B** Tumor size was measured with a caliper on different days. **C** PDX implantation resulted in smaller tumors in anti-TIGIT group mice than in PBS group mice. *P* = 0.0469 by *t*-test, *n* = 7. **D** Percentage of CD8 + T cells in both PDX groups analyzed by flow cytometry. *P* = 0.0275 by *t*-test, *n* = 7. **E** Analysis of the percentage of IFN-γ. *P* = 0.1184 by *t*-test, *n* = 7. **F** Analysis of the percentage of GR2B+. *P* = 0.0034 by *t*-test, *n* = 7. **G** Protein expression of depletion factors TCF-1, TOX1 and NR4A1 after treatment with anti-TIGIT and PBS. **H** Double immune staining pictures of TIGIT/CD8 and CD155/the cell marker of the CD155 expressing cells in the xenograft tumors.

The introduction of ECCA patient tissue into SCID mice, followed by administration of the TIGIT antibody (anti-TIGIT), yielded compelling outcomes. Tumor growth was notably slower, and tumor weight significantly lower in the anti-TIGIT-treated mice in comparison to the control group (*P* < 0.05; Fig. 6A–C). Furthermore, anti-TIGIT treatment prompted an augmentation in effector CD8+ T cells within the tumor tissue (*P* < 0.05; Fig. 6D). The double immune staining showed that TIGIT and CD8 were co-expressed and CD155 and CK-17 were co-expressed in the xenograft tumors (Fig. 6H).

Moreover, the anti-TIGIT treatment group exhibited a pronounced enhancement in the proportion of IFN-γ+ and GRZB+ cells within the CD44+CD8+ T cell subset, relative to the control group (Fig. 6E, F). Notably, the increase in IFN-γ+ cell composition in CD44+CD8+ T cells after anti-TIGIT treatment was not statistically significant when compared to the PBS group (*P* > 0.05). However, a significant increase in the proportion of granzyme B (GRZB) cells within CD44+CD8+ T cells was observed in the anti-TIGIT treatment group (*P* < 0.05 for GRZB+ cells; Fig. 6F).

The results from Western blotting (WB) analyses unveiled an elevation in protein expression of the T cell exhaustion inhibitory factor TCF-1, alongside a reduction in protein expression of TOX1 and NR4A1 in the anti-TIGIT-treated group, as opposed to the PBS group (Fig. 6G). Notably, TCF-1 functions to hinder Tex, while thymocyte selection-associated high mobility group box1 (TOX1) and nuclear receptor subfamily 4 group A member 1 (NR4A1) promote it. These findings collectively suggest that TIGIT antibody treatment may impede ECC growth by counteracting T-cell exhaustion, bolstering the immune capacity and cytotoxic efficacy of CD8+ T cells, thus proposing a promising avenue for ECC treatment.

### TIGIT + CD8 T cells: exhaustion T cells with low potential killing capacity in ECCA

**Fig. 7:**
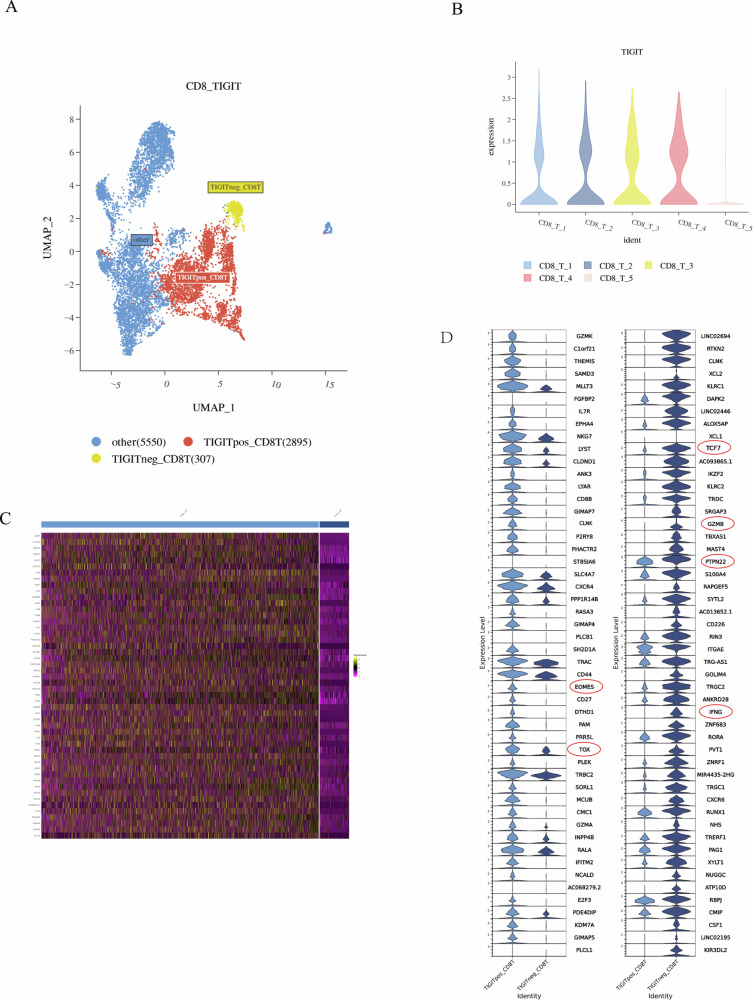
Basic information of the data and the integration of TIGIT+CD8 T and TIGIT−CD8 T cells. **A** UMAP visualizations of TIGIT+CD8 T and TIGIT−CD8+ T cells. **B** The expression of TIGIT in different CD8+ T cell subsets. **C** Heat maps show the top50 differential genes between TIGIT+CD8 T and TIGIT−CD8+ T cells. **D** The violin diagram shows the top50 differential genes between TIGIT+CD8 T and TIGIT−CD8+ T cells.

To further clarify the function of TIGIT+CD8 T and TIGIT−CD8 T subsets, we annotated the subsets according to TIGTI expression level (Fig. 7A), and the expression of TIGIT mainly clustered in CD8 cluster 1–4 (Fig. 7B). Of note, TIGIT+CD8 expressed T cell exhaustion-related genes of TOX, EOMES, and LYAR resembling identified by previous studies, significantly higher than TIGIT-CD8 T cells, while TIGIT−CD8 expressed hindering T cell exhaustion-related genes of TCF7, and PTPN22, significantly higher than TIGIT-CD8 T cells. As well, TIGIT+CD8 expressed potential killing-related genes of GZMB and IFNG (Fig. 7C, D) significantly lower than TIGIT−CD8 T cells. These results imply that TIGIT+CD8 appears to be in a state of exhaustion with low potential killing capacity in ECCA.

## Discussion

In the present study, we performed sing-cell RNA-seq to analyze the immune landscape of TIL isolated from ECCA. Interesting, the results showed T cells are the most abundant cell subpopulation in TILs and may play an important role in the tumor environment of ECCA. Moreover, we found that TIGIT was significantly overexpressed in the TOX+CD8 T cells. Above results suggest that the high expression of TIGIT in ECCA may be involved in T cell exhaustion.

Elevated TIGIT expression has been documented in the tumor microenvironment across various malignancies [7, 8], with TIGIT expression on TILs linked to unfavorable survival outcomes [9]. Our findings corroborate these observations, showcasing that low TIGIT expression aligns with improved patient survival, thus underscoring TIGIT’s potential as a prognostic marker for ECCA. These findings echo earlier research that establishes a connection between increased TIGIT expression in diverse cancers and dismal prognoses [10].

Functional analyses uncovered that TIGIT+CD8+ T cells within ECC patients exhibited markers of T cell exhaustion, such as heightened PD-1 and TIM-3 expression, coupled with diminished TNF-α and IFN-γ production. These observations suggest TIGIT’s active role in driving T cell exhaustion and consequent immune dysfunction, thereby facilitating tumor immune evasion [11]. The immunosuppressive effect of TIGIT might be attributed to its influence on the NF-kB, PI3K, and MAPK pathways [12]. These insights strongly advocate for TIGIT as a potential target for reversing T cell exhaustion [10], thereby augmenting ECCA patients’ antitumor immune responses [13].

Furthermore, the study observed decreased expression of the TCF-1 transcription factor in TIGIT+CD8+ T cells, implying TIGIT’s potential influence on T cell stemness and functionality [14]. Our study found that TCF-1 is negatively correlated with TIGIT. Recent research has indicated that TIGIT is expressed within the exhausted TOX^high^ TCF-1^high^ CD8 T cell subset in both human and murine systems, establishing it as an exhaustion marker [15]. Notably, TCF-1 plays a crucial role in maintaining CD8+ T cells’ stem-like features [14]. This axis curbs T cell exhaustion and upholds T cell stemness, crucial for persistent antiviral CD8+ T cell responses during chronic infections [16].

The study delved further into the therapeutic potential of targeting TIGIT, employing TIGIT antibody treatment in an ECC patient-derived xenograft (PDX) model. The treatment’s outcomes encompassed diminished tumor growth, increased effector CD8+ T cell infiltration, and heightened GRZB production, all indicative of an enhanced immune response and heightened tumor cell eradication. Impressively, the TIGIT antibody treatment successfully counteracted T cell exhaustion, augmenting CD8+ T cell antitumor activity. Multiple studies have demonstrated the efficacy of anti-TIGIT therapy in various mouse models and human cancer patients [7, 17], with promising results even in advanced or metastatic solid tumors [18]. Notably, TIGIT deficiency or blockade has demonstrated protection against multiple myeloma in mice, and heightened effector activity of CD8+ T lymphocytes in myeloma patients [11]. The study’s observation of increased CD8+ T cell abundance following TIGIT antibody treatment may denote heightened immune cell infiltration and activity within the tumor, suggestive of TIGIT antibody’s potential to reverse T cell exhaustion. These CD8+ T cells secrete effector molecules that directly target tumor cells or stimulate other immune cells, amplifying the immune onslaught against tumors. Interestingly, while there was no significant alteration in IFN-γ post-TIGIT antibody treatment, there was a discernible elevation in GRZB production [19], a protease released by natural killer cells in CD8+ cells, highlighting heightened cellular toxicity and lethality in CD8+ cells post TIGIT antibody treatment [20]. Moreover, studies have revealed that certain transcription factors, such as TCF-1 [21], inhibit T cell exhaustion, while others, like TOX1 and NR4A1, promote it. Remarkably, this study showed that TIGIT antibody treatment facilitated TCF-1 expression, concurrently dampening NR4A1 and TOX1, suggesting TIGIT antibody treatment’s potency in reversing T cell exhaustion. The therapeutic mechanism likely involves impeding the interaction between TIGIT and its ligand, consequently alleviating TIGIT’s immunosuppressive effects and elevating CD8+ T cell antitumor activity. Inhibition of TIGIT has exhibited the reversal of cytotoxic T lymphocyte-mediated antitumor immunity depletion, curbing tumor progression in preclinical models [7]. TIGIT expression’s association with tumor advancement underscores its significance [7]. While ongoing large-scale research continues to explore TIGIT antibody treatment, early preclinical outcomes are promising. Human phase I studies involving vibostolimab and etigilimab, both anti-TIGIT antibodies, have demonstrated a favorable safety profile and tolerability across various doses and patient populations [18]. In conclusion, these findings underscore the pivotal role of TIGIT in ECCA’s progression and immune evasion. Targeting TIGIT through antibody therapy emerges as a potent strategy to bolster antitumor immunity and rein in ECCA development. However, comprehensive research and clinical trials are requisite to gauge the complete therapeutic potential, safety, and efficacy of TIGIT antibody treatment in ECCA.

Collectively, the evidence suggests that elevated TIGIT expression in ECCA correlates with poorer prognosis, likely facilitating tumor immune escape by reverting T cell exhaustion and amplifying immunosuppressive pathways. TIGIT antibody therapy holds promise as an immunotherapeutic approach to benefit ECCA patients. Nonetheless, further research is crucial to unveil the intricate mechanisms (TCF-1,Tox1, NR4A1, e.g. regulation axis) through which the TIGIT antibody elicits ECCA remission.

## Supplementary information

full and uncropped western blots

## Data Availability

We confirm we understand the terms of the share upon reasonable request data policy and we will make the data freely available upon request.
